# Regional surface chlorophyll trends and uncertainties in the global ocean

**DOI:** 10.1038/s41598-020-72073-9

**Published:** 2020-09-17

**Authors:** Matthew L. Hammond, Claudie Beaulieu, Stephanie A. Henson, Sujit K. Sahu

**Affiliations:** 1 National Oceanography Centre, European Way, Southampton, SO14 3ZH UK; 2 Ocean and Earth Science, University of Southampton, Southampton, UK; 3 Ocean Sciences Department, University of California Santa Cruz, Santa Cruz, USA; 4 Southampton Statistical Sciences Research Institute, University of Southampton, Southampton, UK

**Keywords:** Climate change, Ocean sciences

## Abstract

Changes in marine primary productivity are key to determine how climate change might impact marine ecosystems and fisheries. Satellite ocean color sensors provide coverage of global ocean chlorophyll with a combined record length of ~ 20 years. Coupled physical–biogeochemical models can inform on expected changes and are used here to constrain observational trend estimates and their uncertainty. We produce estimates of ocean surface chlorophyll trends, by using Coupled Model Intercomparison Project (CMIP5) models to form priors as a “first guess”, which are then updated using satellite observations in a Bayesian spatio-temporal model. Regional chlorophyll trends are found to be significantly different from zero in 18/23 regions, in the range ± 1.8% year^−1^. A global average of these regional trends shows a net positive trend of 0.08 ± 0.35% year^−1^, highlighting the importance of considering chlorophyll changes at a regional level. We compare these results with estimates obtained with the commonly used “vague” prior, representing no independent knowledge; coupled model priors are shown to slightly reduce trend magnitude and uncertainties in most regions. The statistical model used here provides a robust framework for making best use of all available information and can be applied to improve understanding of global change.

## Introduction

Primary production (PP) by phytoplankton comprises approximately half of the global total biospheric production and is vital to most marine ecosystems^1,2^. It is thus important to determine whether phytoplankton abundance is changing and, if so, how rapidly. Chlorophyll-a concentration (chl), a proxy for phytoplankton abundance, is used in studies of trends as it can be measured regularly from satellites at a global scale^3^; it is thus listed as an essential climate variable^4^. As phytoplankton form the base of the marine food web and are mechanistically linked with fishery yield^5^ any changes could have a strong effect on future marine fish stocks^6^.

Biogeochemical models can be used to project future change, as well as to investigate trends over historical periods. Using prescribed atmospheric forcing, a hindcast simulation^7^ has shown a PP decrease of 6.5% over the period of 1960–2006. Modelling projections to the end of the twenty first Century with multiple different parameterizations of marine ecosystems have shown global PP decreases of different magnitude: 2–20%^8^, 8.6%^9^, or 6.5%^10^, each composed of a combination of both PP increases and decreases, varying regionally.

Ocean color satellite records provide the best observational data source for understanding the long-term response of phytoplankton abundance to global climate forcing, due to the data’s large spatial coverage and high temporal resolution^11,12^. However, the short record length and large natural variability of chl can make trend detection challenging^13–16^. Phytoplankton trends estimated from longer time-series tend to show less variability and are of lower magnitude than trends estimated from shorter time-series^17^. Many studies have been conducted on widely available satellite data, although no consensus on the presence/sign of a global phytoplankton abundance trend has been reached yet. Previous studies over different periods and using one or a combination of multiple sensors have reported global trends that were either significantly positive^15,18,19^, negative^20,21^, or not significant^16,22^. It has been suggested that a global average of ~ 30 years of data is required to distinguish a climate change driven chl trend from background variability^13,23^. This suggested record length is substantially longer than the ~ 20 year record available at present, although the global average masks considerable regional variability with some regions requiring less time than the average figure of 30 years.

In order to assess trends in global ocean color data (September 1997–June 2018), a Bayesian spatio-temporal model is used here. In previous work, we demonstrated that this model produces a more accurate fit to chl observations than statistical models that do not account for spatial relationships within the data^24,25^. This approach relies on ‘borrowing strength’, which takes advantage of the fact that trends in chl are likely to be similar at neighbouring grid points^26^. Additionally, this approach provides both a full assessment of uncertainty^24^ and a framework for incorporating information from other sources through a prior distribution. In previous work, however, vague prior distributions have been used to reflect the lack of information about expected trends’ magnitudes and uncertainties^24,25^. As a first guess towards trends in the ocean color record, model output from the IPCC Coupled Model Intercomparison Project (CMIP5) is used here to provide prior information. A combination of Historical and RCP8.5 scenarios from the available CMIP5 model runs are used to form the prior distributions. We also assess the sensitivity of the CMIP5 priors on the resulting trends and uncertainties.

## Results

### Trend estimates and uncertainties with CMIP5 priors

**Figure 1:**
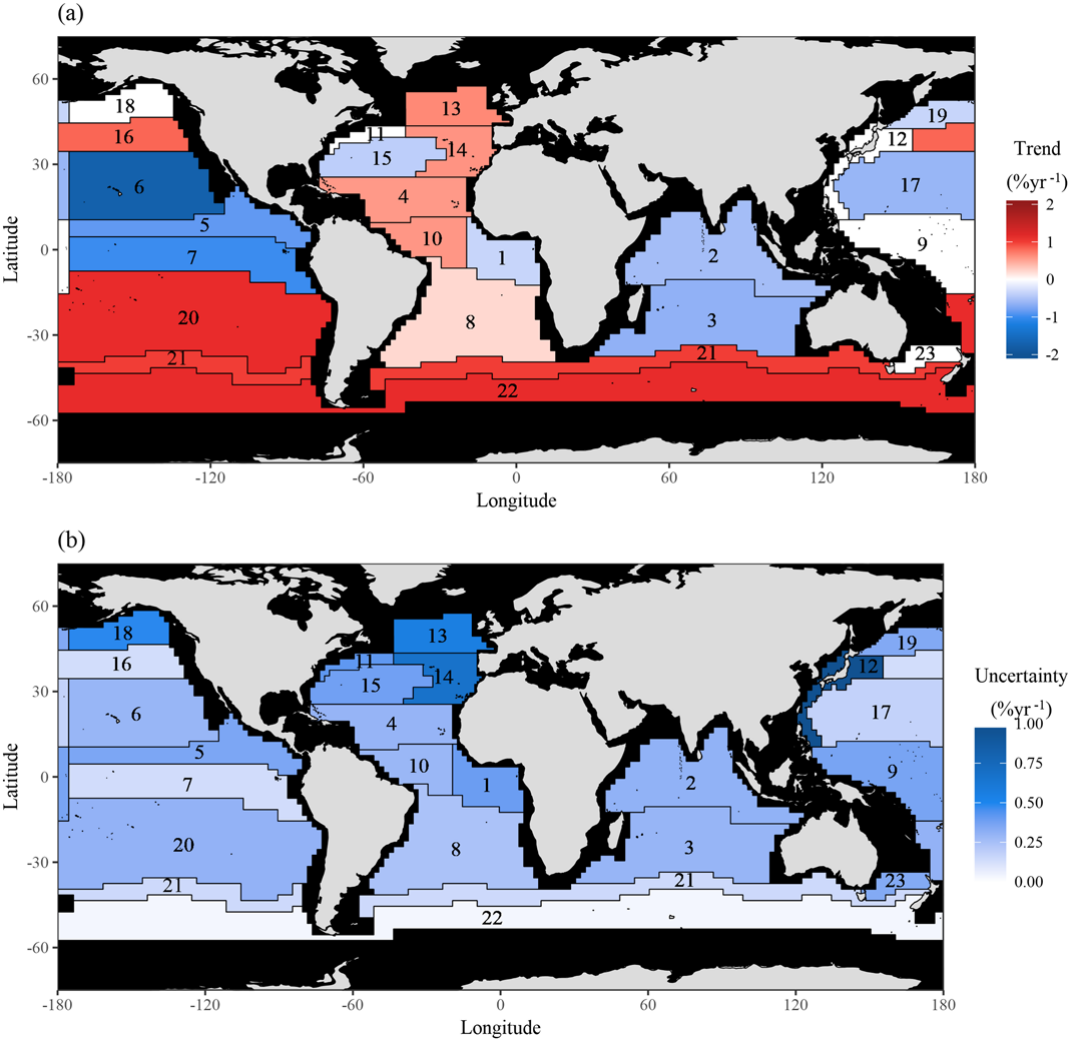
(**a**) The trend estimates and (**b**) their uncertainties (width of 95% HDI) for the space–time model with CMIP5 priors in each region. White regions indicate that the trend is not statistically different from zero. Trends are typically more positive at mid to high latitude. The uncertainty follows a different pattern, appearing to be partially dependent on ocean region; it is high in the North Atlantic and low in the Southern Ocean. Provinces are: (1) Eastern Tropical Atlantic Province, (2) Indian Monsoon Gyres Province, (3) Indian South Subtropical Gyre Province, (4) North Atlantic Tropical Gyral Province, (5) North Pacific Equatorial Countercurrent Province, (6) North Pacific Tropical Gyre Province, (7) Pacific Equatorial Divergence Province, (8) South Atlantic Gyral Province, (9) West Pacific Warm Pool Province, (10) Western Tropical Atlantic Province, (11) Gulf Stream Province, (12) Kuroshio Current Province, (13) North Atlantic Drift Province, (14) North Atlantic Subtropical Gyral Province (East), (15) North Atlantic Subtropical Gyral Province (West), (16) North Pacific Polar Front Province, (17) North Pacific Subtropical Gyre Province (West), (18) Pacific Subarctic Gyres Province (East), (19) Pacific Subarctic Gyres Province (West), (20) South Pacific Subtropical Gyre Province, (21) South Subtropical Convergence Province, (22) Subantarctic Province, and (23) Tasman Sea Province. This map was created by the authors in R v3.4.2 (https://www.r-project.org/) using the ggplot2 v2.2.1 package (https://ggplot2.tidyverse.org/).

The hierarchical model with CMIP5 model priors shows that there is much regional variability in trend estimates and uncertainties (Fig. 1). Note that the uncertainties here are defined based on the width of the 95% Highest Density Interval (HDI). Several regions reveal a negative trend, namely the Indian Ocean (average of − 0.60 ± 0.14% year^−1^) and the majority of the Equatorial and North Pacific (average of − 0.91 ± 0.13% year^−1^). The Atlantic mostly contains regions with positive trends, except the Eastern Tropical Atlantic Province (Region 1) and the North Atlantic Subtropical Gyral Province (West) (Region 15), which show negative trends (of − 0.35 ± 0.19% year^−1^ and − 0.41 ± 0.19% year^−1^, respectively) and the Gulf Stream Province (Region 11) whose trend is not statistically different from zero (0.02 ± 0.21% year^−1^). High latitudes typically display positive trends except in the Northern Pacific Ocean, where the outlook is mixed, specifically the Pacific Subarctic Gyres Province (East) (Region 18) trend is not statistically different from zero (− 0.03% ± 0.24 year^−1^) and the trend in the Pacific Subarctic Gyres Province (West) (Region 19) is negative (− 0.35 ± 0.18% year^−1^). In general, regions where trends are not yet detectable correspond to large uncertainties, such as the Gulf Stream Province (Region 11) and the Pacific Subarctic Gyres Province (East) (Region 18). Trends are statistically different from zero in 18 of the 23 regions we analyse. At the global scale, the statistical model with CMIP5 model priors estimates a weighted average trend of 0.08 ± 0.35% year^−1^ (i.e. suggesting no change). The weighting is based on the average chl and areal extent (in km^2^) of each region. See Table S1 for a complete list of estimated trends and uncertainties, both with and without CMIP5 priors.

### Sensitivity to the choice of priors

**Figure 2:**
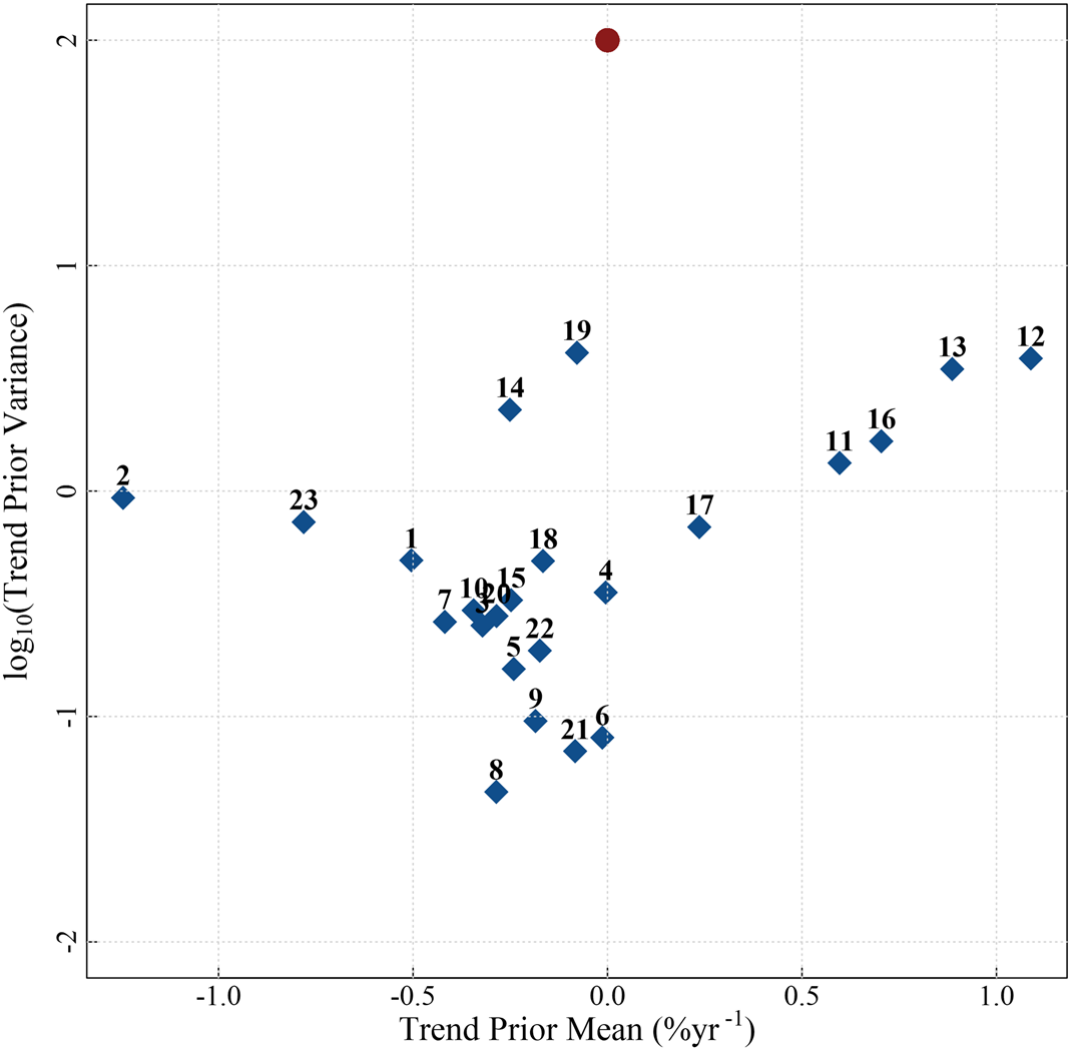
Summary of the CMIP5 prior information: inter-model trend mean and variance from trends fitted on regional time series from each model and ensemble. Trends are estimated from regional average time series for each of the CMIP5 models and ensembles. The red circle indicates the vague prior used, while the blue diamonds indicate the CMIP5 priors for each region. Note that variance is plotted on a logarithmic scale due to the several orders of magnitude difference between the vague priors and the CMIP5 priors. A map of the provinces is provided in Fig. 1 and a list of region names is provided in the caption.

**Figure 3:**
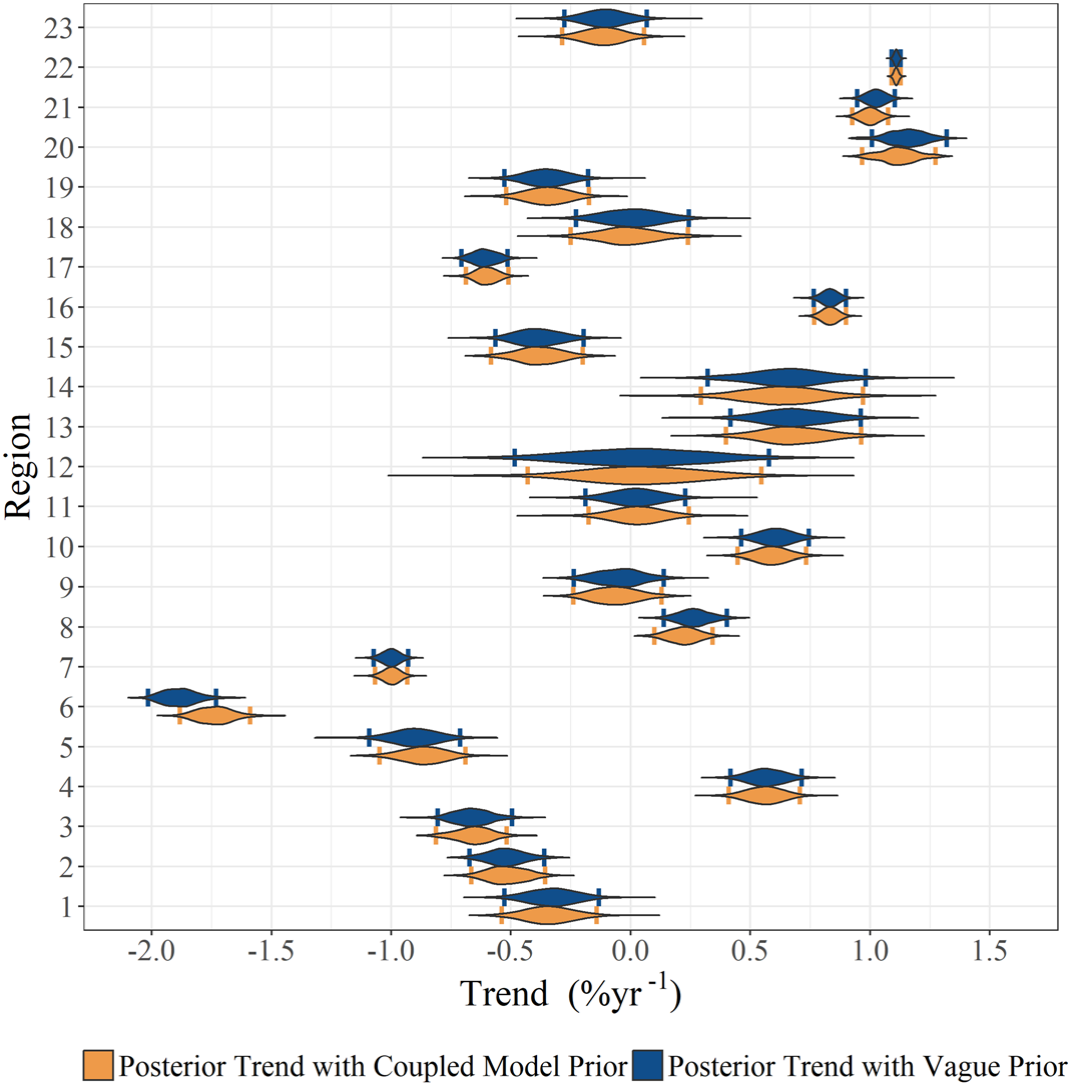
Posterior probability density of the trends for the statistical models with CMIP5 priors and with vague priors. The vertical bars in each probability density mark the upper and lower bounds of the 95% HDI. While the CMIP5 priors may constrain trend estimates and uncertainties in several regions, the trend estimates are not statistically different between using CMIP5 priors and using vague priors (95% HDI). A map of the provinces is provided in Fig. 1 and a list of region names is provided in its caption. The statistical model with CMIP5 model priors yields a global weighted average trend of 0.08% year^−1^.

**Figure 4:**
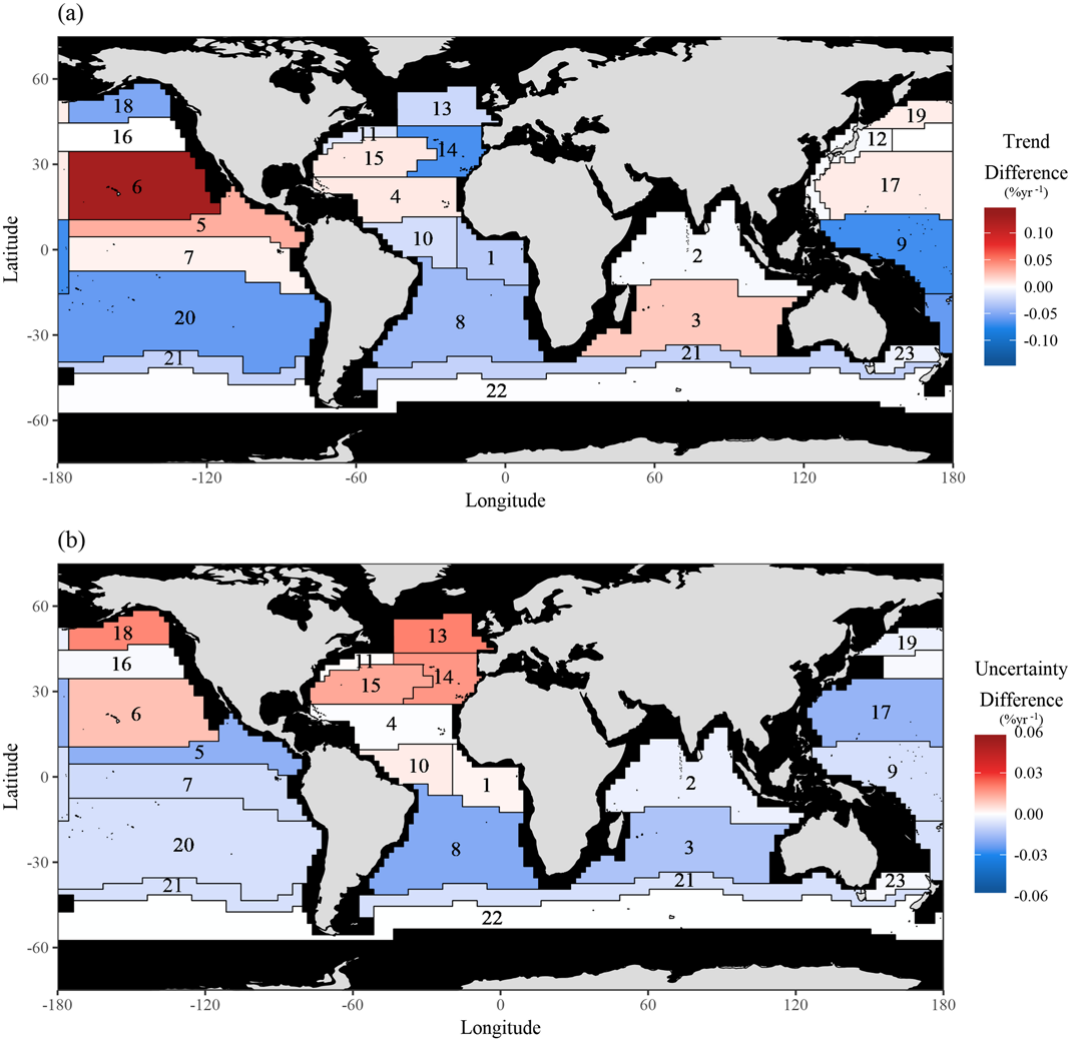
The difference in (**a**) estimated trends and (**b**) their uncertainties, when comparing the models fitted with the CMIP5 priors in each region as opposed to the vague prior. A negative difference indicates that the trend and uncertainty are smaller in the model fitted with the CMIP5 priors. A list of region names is provided in the caption of Fig. 1. This map was created by the authors in R v3.4.2 (https://www.r-project.org/) using the ggplot2 v2.2.1 package (https://ggplot2.tidyverse.org/).

**Figure 5:**
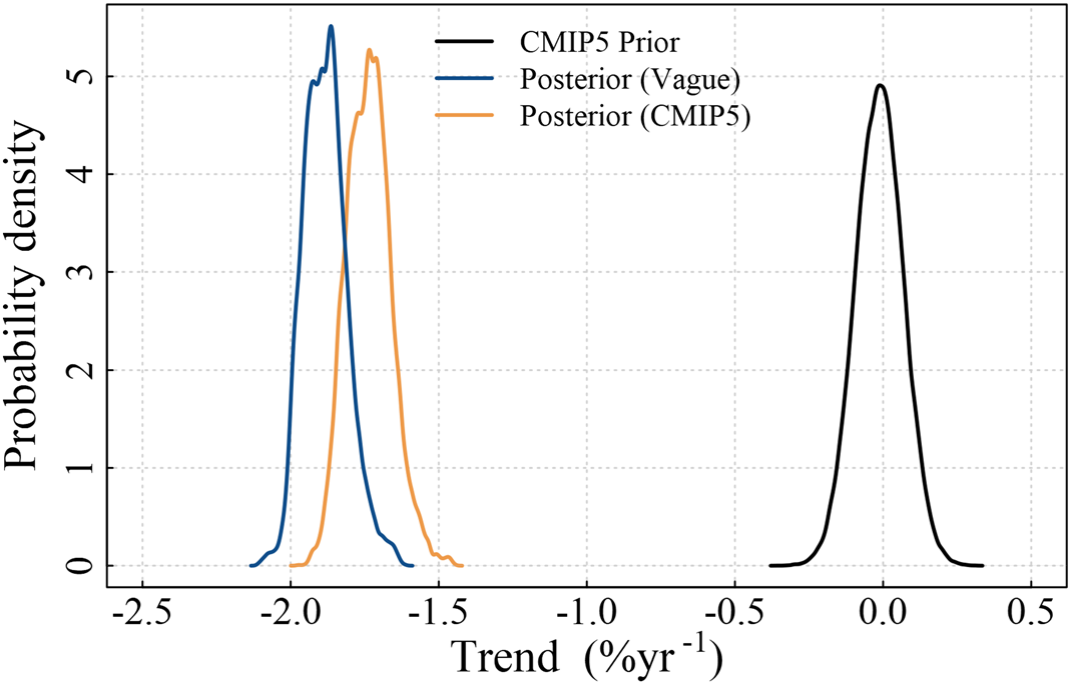
Example of the effect of priors from the North Pacific Tropical Gyre Province (Region 6), the region with the greatest change in trend estimate magnitude. The posterior distribution with CMIP5 priors (orange) is seen to have moved towards zero (and the mode of the CMIP5 prior distribution—black), when compared to the posterior distribution with vague priors (blue). Note that the vague priors are not shown as the distribution is flat over this range. Figure S3 (supporting information) shows distributions from all regions.

The prior information, specifically multi-model means and variances of trends estimated using the CMIP5 models, is shown in Fig. 2 and Table S2. The vague priors have no trend (magnitude of 0.0% year^−1^) and a large variance (100) to reflect the lack of knowledge as to whether trends should be positive or negative, whilst the average of the CMIP5 trends ranges from − 1.2 to 1.1% year^−1^ between regions (the mean across all regions is − 0.089% year^−1^). The variances of the priors obtained from the CMIP5 models are substantially smaller than the vague prior variance (Fig. 2). The effect of the two types of priors is revealed by comparing the posterior trends obtained from fitting the space–time model with CMIP5 and vague priors (Figs. 3, 4). The overall trend in the model with CMIP5 priors is 0.08% year^−1^, while the overall trend in the statistical model with vague priors is 0.094% year^−1^. Table S2 contains a complete list of prior information. This trend reduction highlights that, by introducing CMIP5 prior information, trends tend to become smaller (in 15 of the 23 regions). This reduction in magnitude appears to primarily result from the small variance of the priors (the average variance from all regions is 0.21). The small variance of the CMIP5 prior information, relative to the vague prior, alongside the low magnitude trend of most CMIP5 priors creates an inward pressure on the probability densities, effectively pushing the trends towards zero (Fig. 5). This is despite the fact that the average prior trend magnitude is higher when using the CMIP5 priors (0.04% year^−1^) than when using the vague priors (0.0% year^−1^). The introduction of CMIP5 priors leads to a reduction in trend uncertainty in 15 of the 23 regions (i.e. 65% of regions) (Figs. 3, 4). However there are a few regions where significant increases in uncertainty are seen, typically when CMIP5 trends and observed trends are conflicting, such as in the eastern North Pacific and North Atlantic. However, all these differences are small, and the trend estimates with and without CMIP5 prior information are not deemed statistically different in any region (i.e. their 95% HDIs overlap)—see “Methodology”.

A sensitivity test was performed in order to further analyze the effect of incorporating priors, by fitting models with a range of prior mean and variance values (Tables S3 and S4). Due to computational needs to fit the model, we assess sensitivity for the Tasman Sea Province region (Region 23) only. This example shows that a prior variance of greater than 0.1 has limited effect on trend estimates and essentially no effect on trend uncertainties. Priors with smaller variances have more effect on the trend estimates, reaching a maximum in this sensitivity test when prior variances are 0.001. The CMIP5 priors provide a variance ranging from 0.046 to 4.1, which is still larger than the area of maximum effect identified above as < 0.001. This result may explain why the sensitivity to the choice of priors is generally not that strong. However, it must be noted that there is a degree of variability in these estimates caused by using fewer Markov Chain Monte Carlo (MCMC) iterations in the Bayesian model in this test (10,000 vs 80,000 in the main analysis). A portion of the differences seen in the main analysis may also result from differences between the MCMC chains. Greater consistency between the CMIP5 model trend estimates would be required for them to have greater influence on chl trend estimates as Bayesian priors.

## Discussion

Updated trends in global ocean color data, and their associated uncertainties, are produced using a remotely sensed chl dataset of over 20 years length (covering the period 1997–2018) and a Bayesian spatio-temporal model. For the first time, prior information is provided by chl trends in CMIP5 model output covering the period 1997–2039. A global average weighted trend of 0.08 ± 0.35% year^−1^ is found. This global trend is part of a global pattern of stronger regional trends (± 1.8% year^−1^) that tend to be positive at higher latitudes and are found to be statistically different from zero in 18 of the 23 regions analysed.

Global and regional trends estimated here are on average more positive than a number of previous studies, including a 2.8 × 10^–4^ mgm^−3^ year^−1^ trend using SeaWiFS and MERIS data over the period 1998–2011^15^, as well as a − 0.02% year^−1^ trend using version 2.0 of the ESA OC-CCI dataset covering Sep 1997–Dec 2013^24^. A recent study using SeaWiFS and MODIS data over the period 1998–2015^22^ reported no globally significant trend, albeit within a considerably different statistical framework. Discontinuities within the satellite record are not directly considered here although they may impact trend estimates and their uncertainties^25^. Given the large regional trends of conflicting magnitude and direction, that ultimately lead to a negligible global trend, future studies of chl may wish to focus on the drivers behind regional trends.

For the trends estimated here we provide some explanation of their potential driving factors. Increasing trends in the Southern Ocean may be related to community shifts towards diatoms, a response to increased wind stress and thus mixing; diatoms are typically subject to reduced grazing pressure due to their size^7,22^. A similar increase in the North Atlantic may be explained the same way^7^. Decreases seen in the tropical ocean outside the Atlantic are likely explained by the traditional view of increased stratification leading to increased nutrient limitation and thus decreasing phytoplankton biomass^12,20,27^. It is possible the tropical Atlantic is not undergoing the expected decrease due to a corresponding change in phytoplankton community composition, caused by this increased nutrient limitation. Additionally, internal chl compositions may also be changing in these regions^28–30^, possibly modifying the interpretation. Additional modelling studies, which would ideally also include optical parameterisations, over the same period as the remote sensing record may help further explain some of these trends.

On a regional scale it is interesting to note that the Indian Ocean is estimated to have negative trends in a number of observational studies, although not always over the whole region^12,15,19,21,24,31^. However, it has been estimated^13,23^ that a 40 year continuous record is required to unambiguously distinguish a trend signal from environmental variability here, making it one of the regions requiring the longest record. There is also consistency in the sign of estimated trends in northern hemisphere western subtropical gyres^12,21,22,24,31^, even though these too have been estimated^13^ to require a longer record (37–39 years) in order for a trend to be distinguished from environmental variability.

In other regions of the globe there is typically disagreement in our estimated trends compared to other studies. This disagreement can be explained by both the different statistical approach and the longer record length used in the present study (i.e. more than 20 years). A similar methodology^24^ was used with the ESA OC-CCI v2.0 dataset, which is ~ 5 years shorter than the ESA OC-CCI v3.1 dataset used here, does not include the Visible Infrared Imaging Radiometer Suite (VIIRS) sensor, and has uncorrected decay in MODIS data^32^. The ESA OC-CCI v3.1 dataset also contains two large El Niño events in both 1997/1998 and 2015/2016; the ESA OC-CCI v2.0 dataset only contains the former. Events such as El Niño can have significant effects on trend estimates, and thus it is important to have as long a record length as possible, so as to minimize their effect on trend estimates^33^. To assess the effect of record length we have included a comparison of trends detected over the periods of September 1997–December 2016 and September 1997–December 2013 with the longest period used here ending in December 2018 with the same methodology (see Text S2 and Figure S2 in supporting information). Trends detected over 1997–2016 and 1997–2018 are similar in sign and magnitudes in most regions, while there are discrepancies with the 1997–2013 trends (Figure S2). The consistency in the sign and magnitude of estimated trends between the two longest observational records suggest trends may be becoming robust with reduced influence from natural variability in some regions. It should be noted that other studies have found that trends in remotely sensed reflectance emerge from natural variability earlier than chl trends, and thus could be a focus for future studies using spatio-temporal statistics for trend detection^30^.

We have used CMIP5 models to form prior distributions for chl trends to reflect independent knowledge from observations. CMIP5 trends are found to be typically of lower magnitude than the observational trends. This difference may result from either the longer time-series used for the CMIP5 trends, allowing interannual variability to be more successfully isolated when estimating trends, or CMIP5 models underestimating trends. However, the models produce a relatively wide range of trend estimates, meaning that these priors reflect large differences between models. Weighting or other selection approaches could be used to reduce the inter-model uncertainty, which could potentially allow for better constraints on trend estimates in the statistical model.

Another potential route for increasing the impact of priors, may be by using further information from the CMIP5 models, currently only trend estimate information is used as a prior. However, as this forms only one of the number of priors used by the model, this will effectively limit the degree of constraint on the model. Instead additional prior information (e.g. the intercept, seasonality magnitude, and potentially other hyperparameters) from CMIP5 models could be used. However, care should be taken to ensure that these priors are realistic so as not to produce an increased uncertainty of trend estimates by their inclusion.

The Bayesian spatio-temporal model provides a promising and robust framework for studies of climate change driven trends in space–time datasets with limited coverage by ‘borrowing strength’. Furthermore, multiple sources of information (e.g. models and observations) can be used to improve estimates of climate change driven trends and constrain their uncertainties which is essential for policy making decisions supporting marine ecosystems and fisheries. Additional improvements of our updated estimates could be made by incorporating in situ data (where available) in the hierarchical model or as Bayesian priors to further reduce their variance.

## Methods

### Data

The chl data are sourced from the ESA OC-CCI v3.1 product^34^ (available at: https://www.esa-oceancolour-cci.org/). This dataset combines the SeaWiFS, MERIS, MODIS, and VIIRS sensors using band-shifting and bias-correction techniques to create a monthly time-series from September 1997 to June 2018 inclusive. The importance of the length of time period, and its effect on trend estimates, is discussed fully in Supporting Information Text S2. The data is downscaled to a 1° grid by averaging within 1° boxes. Supporting Figure S5 and Table S5 compare results between this approach and an equivalent approach using an equal area 100 km grid, but only minimal differences are found.

Model data comes from CMIP5 models (sourced from https://esgf-node.llnl.gov/projects/cmip5/), see Table 1 for a full list of models used. Models and ensembles with available monthly chl output, run under the RCP8.5 and historical scenarios, were used. RCP8.5 and historical outputs are joined, omitting data outside the period of interest, to create a continuous dataset covering September 1997–April 2039 (i.e. twice the current length of the observational period) and starting at the point when SeaWiFS became operational. This selection provides a record length of ~ 42 years, sufficient in most areas of the globe for climate change driven chl trends to become distinguishable from background interannual variability^13^. A comparison of the use of different time periods to determine the priors can be found in the supplementary information (Text S1 and Figure S1). A (natural) log-transformation of the chl data is used, for both observational and model output^35^. While trends are estimated as log differences per month, the reported trends have been converted so that percentage changes should be considered as percentage changes in un-transformed chl.

**Table 1:** Models used, their marine biogeochemical component, associated references, and number of ensemble runs.

Model names	Bioegeochemical model	References	Number of ensembles
CMCC-CESM	PELAGOS	^36^	1
CNRM-CM5	PISCES	^37,38^	1
GFDL ESM2G	TOPAZ2	^39^	1
GFDL ESM2M	TOPAZ2	^39^	1
GISS E2 H CC	NOBM	^40^	1
GISS E2 R CC	NOBM	^40^	1
HadGEM2 CC	Diat-HadOCC	^41^	3
HadGEM2 ES	Diat-HadOCC	^41^	4
IPSL CM5A LR	PISCES	^37,38^	4
IPSL CM5A MR	PISCES	^37,38^	1
IPSL CM5B LR	PISCES	^37,38^	1
MPI ESM LR	HAMOCC5.2	^42^	3
MPI ESM MR	HAMOCC5.2	^42^	1
MRI ESM1	MRI.COM3	^43^	1

Trends in 23 open ocean regions are analyzed, with boundaries defined as by Longhurst^44,45^. The province approach was chosen as these regions are defined by characteristic physical forcing and biogeochemical factors, so as to produce regions with as similar trends as possible. This division is necessary as the model formulation necessitates producing one estimate in each region, see model formulation below. Coastal and polar waters were omitted due to issues with data availability and quality.

### Model formulation

In each of the 23 Longhurst regions selected for study a hierarchical Bayesian spatio-temporal model is fitted. Equation (1) represents the first level or data level, where the relationship between the observed chl ${Z}_{n,t}$, at location $n=1, 2, \dots ,N$ (where N is the total number of 1° grid cells in each region and ranges between 116 and 3,785) and at month $t=1, 2, \dots , 250$, and its true value ${O}_{n,t}$ and random measurement error ${\varepsilon }_{n,t}$ is stated:

1 $${Z}_{n,t}={O}_{n,t}+\boldsymbol{ }{\varepsilon }_{n,t}$$

The true value is represented by the following assumed regression model:

2 $${O}_{n,t}={{\varvec{x}}}_{n,t}^{^{\prime}}{\varvec{\beta}}+\boldsymbol{ }{{\varvec{a}}}_{n}^{\boldsymbol{^{\prime}}}{{\varvec{w}}}_{m,t}$$

where ${{\varvec{x}}}_{n,t}$ represents the covariates and intercept, ${\varvec{\beta}}$ represents the regression coefficients (detailed below), and the term ${{\varvec{a}}}_{n}^{\boldsymbol{^{\prime}}}{{\varvec{w}}}_{m,t}$ represents spatial and temporal correlation. The spatial correlation, which considers the relationship between points dependent on their distance in km, is represented by an exponential decay away from site $n$, with spatial correlation becoming completely negligible by approximately 1,500 km^25^. The temporal correlation is represented by an AR(1) process (i.e. dependent on the preceding month only). The selected covariates are: the time of the observation (to represent the trend) and a seasonality term. The seasonality term is represented sinusoidally as follows:

3 $${\beta }_{\mathit{seas}}\mathrm{cos}\left(\frac{2\pi t}{T}+\varphi \right)$$

with amplitude (${\beta }_{seas}$) fitted by the regression model, the period (T) fixed at 12 months (i.e. annually), and phase ($\varphi$) selected before fitting the regression model so that the peak of the cycle corresponds with the regional average month of peak chl during the year.

Underlying these equations are the prior distributions which can be used to represent existing understanding, or left vague to represent no clear previous understanding (i.e. a vague prior). The prior distribution used here for the trend is represented by a normal distribution with a specified mean and variance. For the vague prior, a mean trend of 0% year^−1^ and a large variance (100) are used. The prior based on CMIP5 model output combines RCP8.5 and historical scenarios over 1997–2039 to completely cover the observational period and allow a sufficiently long record to distinguish trends from interannual variability. To form the CMIP5 priors, a generalized least squares linear regression model with temporal correlation assuming an AR(1) process and no spatial correlation is fitted to the average time series in each Longhurst region for each CMIP5 model and ensemble. Spatial correlation terms were omitted, for the model trend estimates only, due to the high computational cost resulting from the longer time-series and number of models. Omitting these terms is expected to result in a loss of useful information, although the longer time-series should compensate for this. Individual ensembles are averaged per model, to avoid adding additional weight to individual models, before a multi-model mean and variance is calculated to provide information for a prior. A complete list of prior trend estimates is provided in Table S2.

This study is focused on the regression coefficient for the trend from the above Bayesian statistical model, which we estimate as the mode of the posterior distribution. The uncertainty of the trend parameter is represented by the 95% credible interval which is defined as the 95% HDI^46^. To assess the effect of introducing the CMIP5 prior information, results for the statistical model with the CMIP5 priors are compared to results from a statistical model with vague priors. To evaluate whether trends are likely to be different in the two scenarios their 95% credible intervals are compared to determine if there is an overlap.

The model fit was estimated in R using the spTimer package^46^ using 80,000 MCMC iterations following burn-in to allow for good MCMC convergence. Full details on the package and model setup can be found in previous works^24,47^.

## Supplementary information

Supplementary Information.
