# Supplementary material for: Regional surface chlorophyll trends and uncertainties in the global ocean
Source: Sci Rep. 2020 Sep 17;10:15273. doi: 10.1038/s41598-020-72073-9 (PMC7498587; doi:10.1038/s41598-020-72073-9)
Supplement: Supplementary file 1 — Supplementary Information. [file 41598_2020_72073_MOESM1_ESM.docx]

Supplementary information for:

**Regional Surface Chlorophyll Trends and Uncertainties in the Global Ocean**

Matthew L. Hammond^1,2^, Claudie Beaulieu^2,3^, Stephanie A. Henson^1^ & Sujit K. Sahu^4^

1. National Oceanography Centre, Southampton
2. Ocean and Earth Science, University of Southampton
3. Ocean Sciences, University of California Santa Cruz
4. Southampton Statistical Sciences Research Institute, University of Southampton

**List of Text:**

Text S1: Effect of the time period used to determine CMIP5 Priors 7

Text S2: Effect of the observational time period used to estimate trends 9

**List of Tables:**

Table S1: Full list of trends and credible intervals 2

Table S2: Full list of prior information trends 3

Table S3: Sensitivity of the best trend estimate to prior mean and variance 4

Table S4: Sensitivity of the credible interval width to prior mean and variance 5

Table S5: Comparison of posterior trend distributions (1° vs 100km data grids) 6

**List of Figures:**

Figure S1: Comparison of the time period used to determine CMIP5 Priors 8

Figure S2: Comparison of observational time periods used to estimate trends 10

Figure S3: Comparison of posterior and prior trend distributions from all regions 11-12

Figure S4: Regional average time-series 13-14

Figure S5: Comparison of posterior trend distributions (1° vs 100km data grids) 15

Table S1. Full list of modal trends and their 95 % credible interval (CI), for the statistical model with vague priors, and the statistical model with CMIP5 priors. See Figure 1 for region names.

|  | Model with Vague Priors | | | | Model with CMIP5 Priors | | |
| --- | --- | --- | --- | --- | --- | --- | --- |
| Region | Trend Value  (% yr ^-1^) | Lower CI Boundary  (% yr ^-1^) | Upper CI Boundary  (% yr ^-1^) | Trend Value  (% yr ^-1^) | | Lower CI Boundary  (% yr ^-1^) | Upper CI Boundary  (% yr ^-1^) |
| 1 | -0.31 | -0.53 | -0.13 | -0.35 | | -0.54 | -0.14 |
| 2 | -0.54 | -0.67 | -0.36 | -0.55 | | -0.67 | -0.36 |
| 3 | -0.67 | -0.81 | -0.49 | -0.65 | | -0.81 | -0.52 |
| 4 | 0.56 | 0.42 | 0.72 | 0.57 | | 0.41 | 0.71 |
| 5 | -0.9 | -1.1 | -0.71 | -0.87 | | -1.0 | -0.69 |
| 6 | -1.9 | -2.0 | -1.7 | -1.7 | | -1.9 | -1.6 |
| 7 | -1.0 | -1.1 | -0.93 | -0.99 | | -1.1 | -0.93 |
| 8 | 0.26 | 0.14 | 0.40 | 0.22 | | 0.099 | 0.34 |
| 9 | -0.0063 | -0.24 | 0.14 | -0.076 | | -0.24 | 0.13 |
| 10 | 0.60 | 0.46 | 0.74 | 0.58 | | 0.45 | 0.73 |
| 11 | 0.032 | -0.19 | 0.23 | 0.017 | | -0.18 | 0.24 |
| 12 | 0.029 | -0.48 | 0.58 | 0.026 | | -0.43 | 0.55 |
| 13 | 0.68 | 0.42 | 0.96 | 0.66 | | 0.40 | 0.96 |
| 14 | 0.67 | 0.32 | 0.98 | 0.60 | | 0.29 | 0.97 |
| 15 | -0.42 | -0.56 | -0.20 | -0.41 | | -0.58 | -0.20 |
| 16 | 0.83 | 0.76 | 0.90 | 0.83 | | 0.77 | 0.90 |
| 17 | -0.62 | -0.71 | -0.51 | -0.61 | | -0.69 | -0.51 |
| 18 | 0.025 | -0.23 | 0.24 | -0.028 | | -0.25 | 0.24 |
| 19 | -0.36 | -0.53 | -0.18 | -0.35 | | -0.52 | -0.17 |
| 20 | 1.2 | 1.0 | 1.3 | 1.1 | | 0.97 | 1.3 |
| 21 | 1.0 | 0.95 | 1.1 | 1.0 | | 0.93 | 1.1 |
| 22 | 1.1 | 1.1 | 1.1 | 1.1 | | 1.1 | 1.1 |
| 23 | -0.11 | -0.28 | 0.067 | -0.12 | | -0.29 | 0.057 |

Table S2. Full list of modal trends and their 95 % HDI boundaries, for the CMIP5 models. See Figure 1 for region names.

| Region | Trend Value  (% yr ^-1^) | Lower HDI Boundary  (% yr ^-1^) | Upper HDI Boundary  (% yr ^-1^) |
| --- | --- | --- | --- |
| 1 | -0.50 | -1.5 | 0.49 |
| 2 | -1.2 | -3.1 | 0.56 |
| 3 | -0.32 | -0.81 | 0.18 |
| 4 | -0.0044 | -0.70 | 0.69 |
| 5 | -0.24 | -0.56 | 0.076 |
| 6 | -0.013 | -0.17 | 0.14 |
| 7 | -0.42 | -0.94 | 0.079 |
| 8 | -0.29 | -0.38 | -0.20 |
| 9 | -0.18 | -0.38 | -0.00071 |
| 10 | -0.34 | -0.91 | 0.24 |
| 11 | 0.6 | -2.0 | 3.2 |
| 12 | 1.1 | -6.4 | 8.7 |
| 13 | 0.89 | -6.1 | 7.5 |
| 14 | -0.25 | -4.8 | 4.2 |
| 15 | -0.25 | -0.89 | 0.40 |
| 16 | 0.70 | -2.5 | 4.0 |
| 17 | 0.24 | -1.1 | 1.6 |
| 18 | -0.17 | -1.1 | 0.77 |
| 19 | -0.079 | -8.3 | 7.9 |
| 20 | -0.29 | -0.84 | 0.25 |
| 21 | -0.083 | -0.22 | 0.053 |
| 22 | -0.17 | -0.55 | 0.21 |
| 23 | -0.78 | -2.2 | 0.64 |

Table S3. Example sensitivity analysis showing the effect, on the best trend estimate, of a range of mean and variance values in the trend prior distribution. This example is for the Tasman Sea Province region (Region 23), although similar results are found in other regions. The trend estimate approaches the prior mean in a manner inversely proportional to the prior variance. The table reports trend values in % yr ^-1^, calculated in units of log(mg m^-3^) per month.

|  | Prior Variance | | | | | | | | | |  |
| --- | --- | --- | --- | --- | --- | --- | --- | --- | --- | --- | --- |
|  | 0.0001 | 0.001 | | 0.01 | | 0.1 | 1 | 10 | 100 | | |
| Prior Mean (% yr ^-1^) | Trend Estimate (% yr ^-1^) | | | | | | | | |  |  |
| -5 | -4.9 | -4.5 | -2.4 | | -0.41 | | -0.017 | 0.014 | 0.039 | | |
| -1 | -0.99 | -0.90 | -0.46 | | -0.051 | | 0.032 | 0.039 | 0.031 | | |
| -0.5 | -0.49 | -0.45 | -0.22 | | -0.004 | | 0.034 | 0.034 | 0.051 | | |
| -0.1 | -0.097 | -0.089 | -0.033 | | 0.024 | | 0.047 | 0.038 | 0.025 | | |
| -0.05 | -0.049 | -0.039 | -0.014 | | 0.025 | | 0.028 | 0.036 | 0.017 | | |
| -0.01 | -0.0089 | -0.0089 | 0.010 | | 0.028 | | 0.027 | 0.047 | 0.023 | | |
| 0 | -0.00095 | 0.0033 | 0.0075 | | 0.022 | | 0.047 | 0.036 | 0.021 | | |
| 0.01 | 0.010 | 0.016 | 0.021 | | 0.035 | | 0.026 | 0.051 | 0.034 | | |
| 0.05 | 0.049 | 0.053 | 0.039 | | 0.033 | | 0.045 | 0.038 | 0.039 | | |
| 0.1 | 0.098 | 0.094 | 0.074 | | 0.056 | | 0.051 | 0.040 | 0.040 | | |
| 0.5 | 0.49 | 0.45 | 0.25 | | 0.075 | | 0.028 | 0.031 | 0.038 | | |
| 1 | 0.99 | 0.91 | 0.50 | | 0.12 | | 0.063 | 0.045 | 0.034 | | |
| 5 | 4.9 | 4.5 | 2.4 | | 0.46 | | 0.065 | 0.051 | 0.037 | | |

Table S4. Example sensitivity analysis showing the effect, on the trend uncertainty, of a range of mean and variance values in the trend prior distribution. This example is from the Tasman Sea Province region (Region 23), although similar results are found in other regions. The prior mean does not affect the trend uncertainty. The uncertainty appears to decrease proportionally with the prior variance. However prior variances of greater than 0.1 seem to have a relatively limited effect on the trend credible interval width. The table reports credible interval widths in % yr ^-1^, calculated in units of log(mg m^-3^) per month.

|  | Variance | | | | | | | | | | | | |
| --- | --- | --- | --- | --- | --- | --- | --- | --- | --- | --- | --- | --- | --- |
|  | 0.0001 | 0.001 | | 0.01 | | 0.1 | | 1 | | 10 | | 100 | |
| Prior Mean (% yr ^-1^) | Trend Uncertainty (% yr ^-1^) | | | | | | | | | | | | |
| -5 | 0.039 | 0.12 | 0.28 | | 0.36 | | 0.37 | | 0.37 | | 0.37 | |  |
| -1 | 0.039 | 0.11 | 0.27 | | 0.36 | | 0.37 | | 0.36 | | 0.36 | |  |
| -0.5 | 0.039 | 0.12 | 0.27 | | 0.35 | | 0.37 | | 0.36 | | 0.37 | |  |
| -0.1 | 0.039 | 0.12 | 0.27 | | 0.35 | | 0.37 | | 0.37 | | 0.38 | |  |
| -0.05 | 0.039 | 0.12 | 0.27 | | 0.36 | | 0.39 | | 0.37 | | 0.38 | |  |
| -0.01 | 0.039 | 0.12 | 0.27 | | 0.36 | | 0.37 | | 0.37 | | 0.37 | |  |
| 0 | 0.038 | 0.12 | 0.27 | | 0.35 | | 0.37 | | 0.37 | | 0.37 | |  |
| 0.01 | 0.039 | 0.12 | 0.27 | | 0.36 | | 0.37 | | 0.37 | | 0.37 | |  |
| 0.05 | 0.039 | 0.12 | 0.27 | | 0.36 | | 0.36 | | 0.37 | | 0.38 | |  |
| 0.1 | 0.039 | 0.12 | 0.27 | | 0.36 | | 0.38 | | 0.38 | | 0.37 | |  |
| 0.5 | 0.039 | 0.12 | 0.27 | | 0.35 | | 0.37 | | 0.36 | | 0.37 | |  |
| 1 | 0.039 | 0.12 | 0.27 | | 0.37 | | 0.38 | | 0.37 | | 0.37 | |  |
| 5 | 0.039 | 0.12 | 0.27 | | 0.35 | | 0.36 | | 0.36 | | 0.38 | |  |

Table S5. The effect of data gridding on summary values of the posterior distributions of the trend estimate. Only minimal differences are seen between the two posterior distributions (full distributions are plotted in Figure S5).

|  | 1° grid | | | | 100km grid | | |
| --- | --- | --- | --- | --- | --- | --- | --- |
| Region | Trend Value  (% yr ^-1^) | Lower CI Boundary  (% yr ^-1^) | Upper CI Boundary  (% yr ^-1^) | Trend Value  (% yr ^-1^) | | Lower CI Boundary  (% yr ^-1^) | Upper CI Boundary  (% yr ^-1^) |
| 14 | 0.67 | 0.32 | 0.98 | 0.64 | | 0.32 | 1.0 |

**Text S1: Effect of the time period used to determine CMIP5 priors**

Model prior information is determined using time series analysis on the CMIP5 data for each model and ensemble, to determine trends and inter-model uncertainty of the trend. A certain time period of the model must be defined to determine the model prior information. In the main article the time period starts in September 1997, i.e. the start of the observational period, and runs to April 2039, thus having twice the length of the observational period, i.e. ~42 years, meaning that the length is sufficient in most areas of the globe for a climate change driven chl trend to become distinguishable from background interannual variability^13^.

To investigate the effect of the time period on the trend values used as priors, a comparison is made here of the time period from the main article and two others; one solely covering the observational period (i.e. September 1997 to June 2018), and one covering three times the length of the observational period (i.e. starting in November 1976 and ending in April 2039). From this comparison one can see that the CMIP5 trends are exclusively negative in the case of the longer time period with only a few being positive in the case of the shorter time periods (Figure S1). Note that this is also different to the observational trends in a number of regions (see Figure 2), where positive trends can instead be seen. The differences between the approaches (and the observational trends) are likely caused by decadal variability or the inclusion of the period prior to 1997. The differences in trends between models for the shortest period (i.e. the same as the observational record length) and observations could be down to two reasons: the model representations of biogeochemistry & physics and the differences in the statistical model used to estimate trends. When estimating trends in the model data spatial correlation was ignored (it was included when analyzing the observational data) this has been previously shown to lead to more negative trends over a similar time period^24^. It should also be noted that the longest time period has the least inter-model uncertainty, with the shortest generally having the most, although not in all regions (which may be being affected by decadal variability). This will mean that using a longer time period will produce priors that will have a greater effect on the trend estimates from the Bayesian model (Table S3).


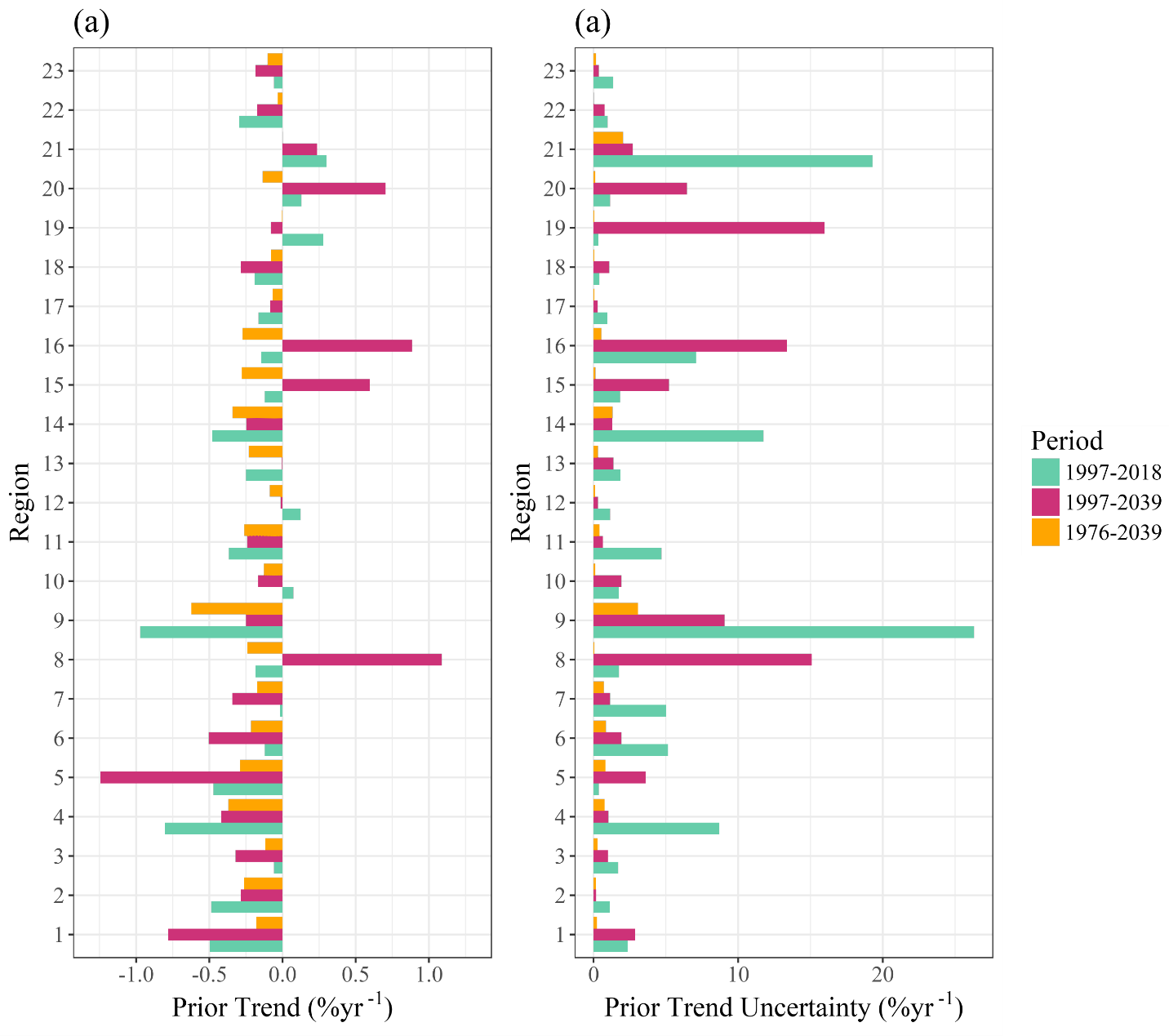


Figure S1. A comparison of the effect of the time period on the CMIP5 priors.

**Text S2: Effect of the observational time period used to estimate trends**

The time period used to estimate trends has been shown to be of significant importance, with trends estimated from longer time-series tending to show less variability and lower magnitude when compared to trends estimated from shorter time-series^17^. In order to demonstrate that the longer time series of over 20 years used here is of sufficient length, a comparison is made of trends estimated using three time series of different length (Figure S2). Three different time periods are shown: September 1997 to June 2018, September 1997 to December 2016, and September 1997 to December 2013. The longest two time periods show very similar trends albeit with some slight differences in trend magnitudes. However, the shortest time period shows a large number of differences to the two longer periods, with some trends switching sign. The regions which are most affected by the different record length appear to be primarily in the equatorial and southern Atlantic and Pacific. These changes likely relate to any uncorrected decay in data from MODIS^28^ and/or strong interannual variability. The September 1997 to December 2013 time period begins with a very strong El Niño event. A similarly large event is not seen again until 2016, which is only present in the two longer datasets. By including the two events the longer time periods are less affected by interannual variability. This comparison highlights the importance of a longer record, to help minimize the effects of interannual variability, and that the long time period used here appears to successfully mitigate these effects.


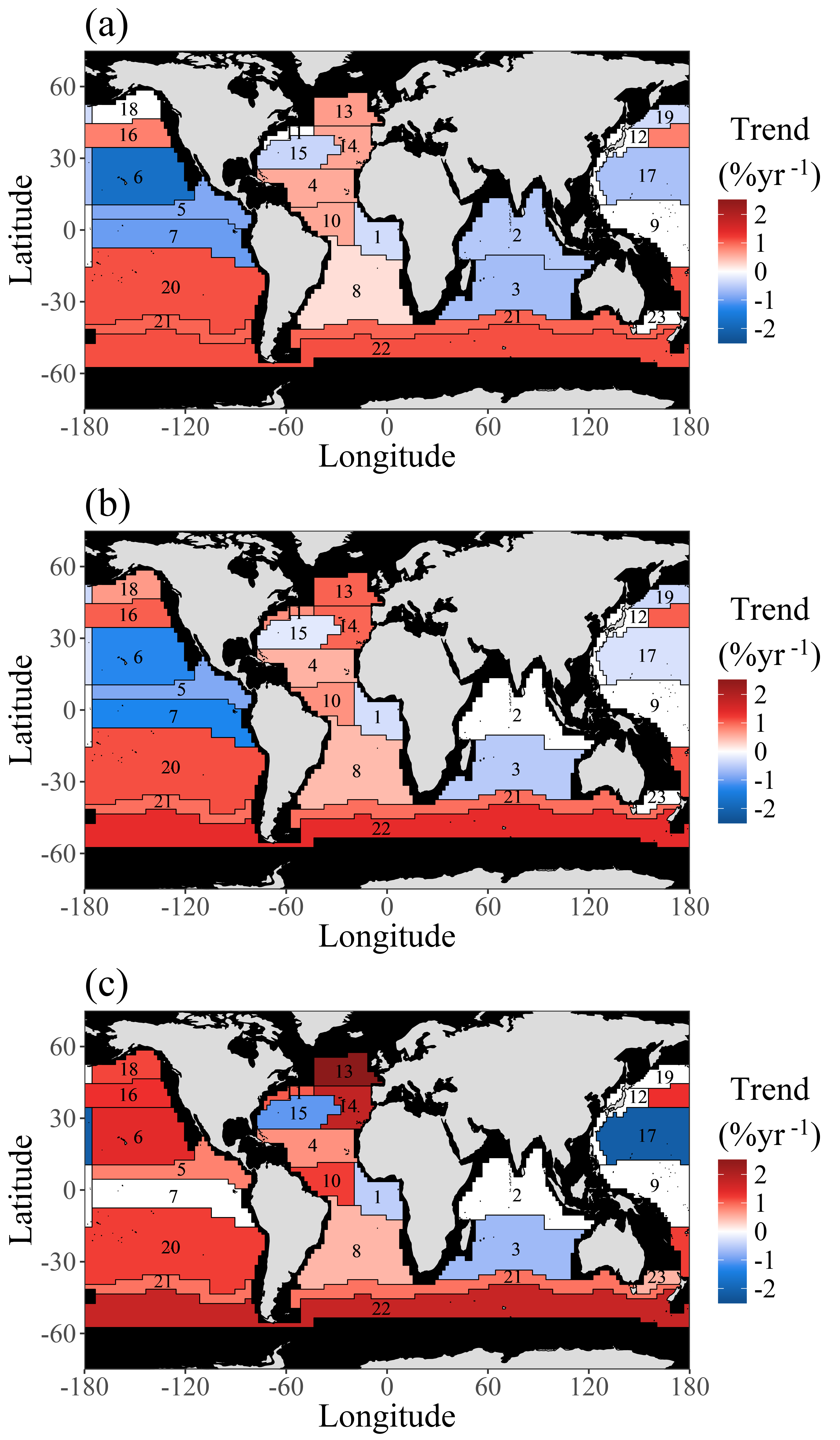


Figure S2. A comparison of the effect of observational time period on estimated trends. (a) shows the same time period as used in the main article (September 1997 to June 2018), (b) shows the time period September 1997 to December 2016, and (c) shows the time period September 1997 to December 2013. This map was created by the authors in R v3.4.2 (https://www.r-project.org/) using the ggplot2 v2.2.1 package (https://ggplot2.tidyverse.org/).


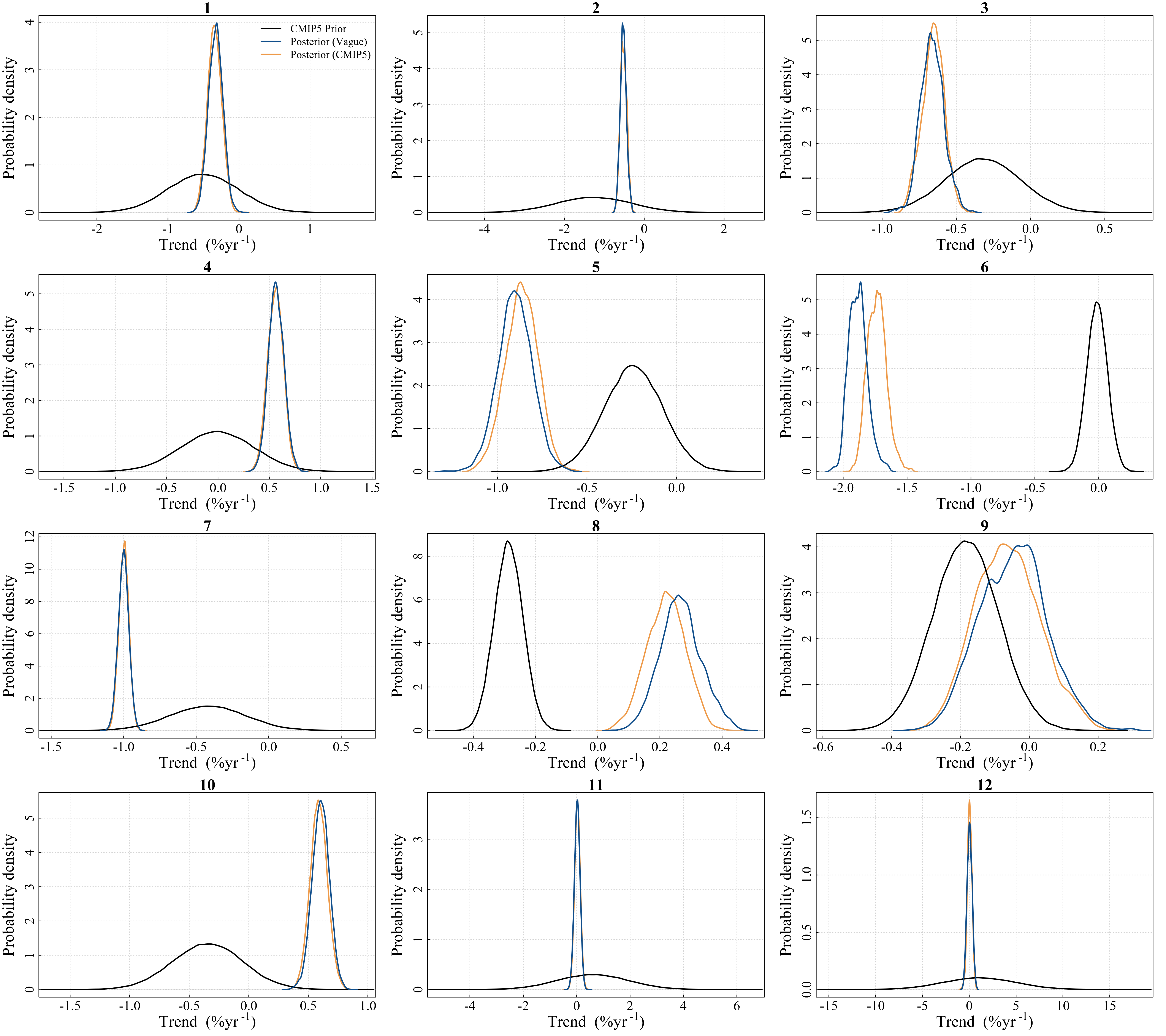


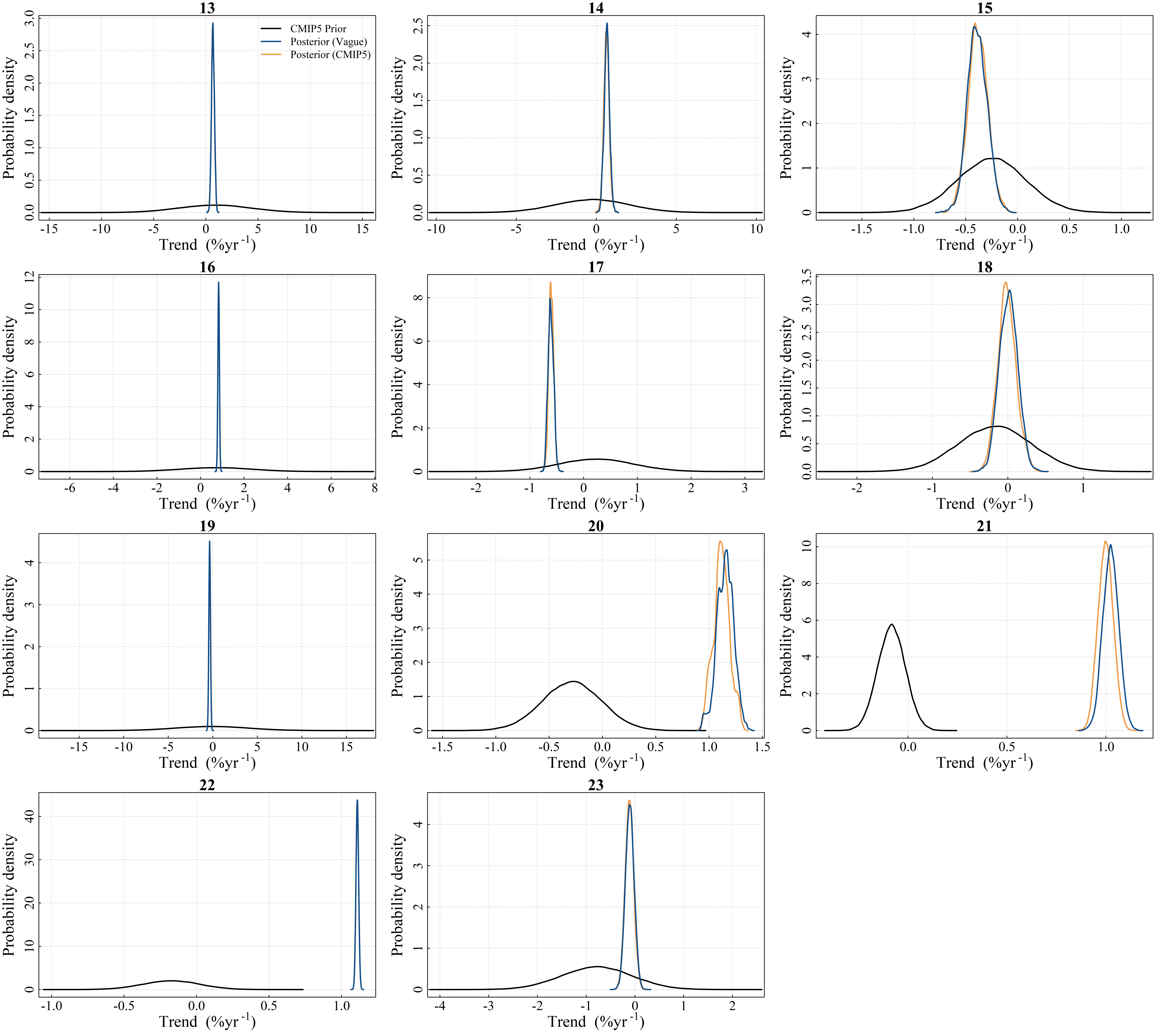


Figure S3. The effect of priors on the posterior distributions. The posterior distribution with CMIP5 priors (orange) is seen to move towards the mode of the CMIP5 prior distribution (black), when compared to the posterior distribution with vague priors (blue). Note that the vague priors are not shown as the distribution is flat over this range. See the caption of Figure 1 (main text) for region names.








Figure S4. Regional average time-series, displaying example variability (both seasonal and inter-annual within each region). Note that by averaging over the entire region these images will underestimate the true range of variability. See the caption of Figure 1 (main text) for region names.


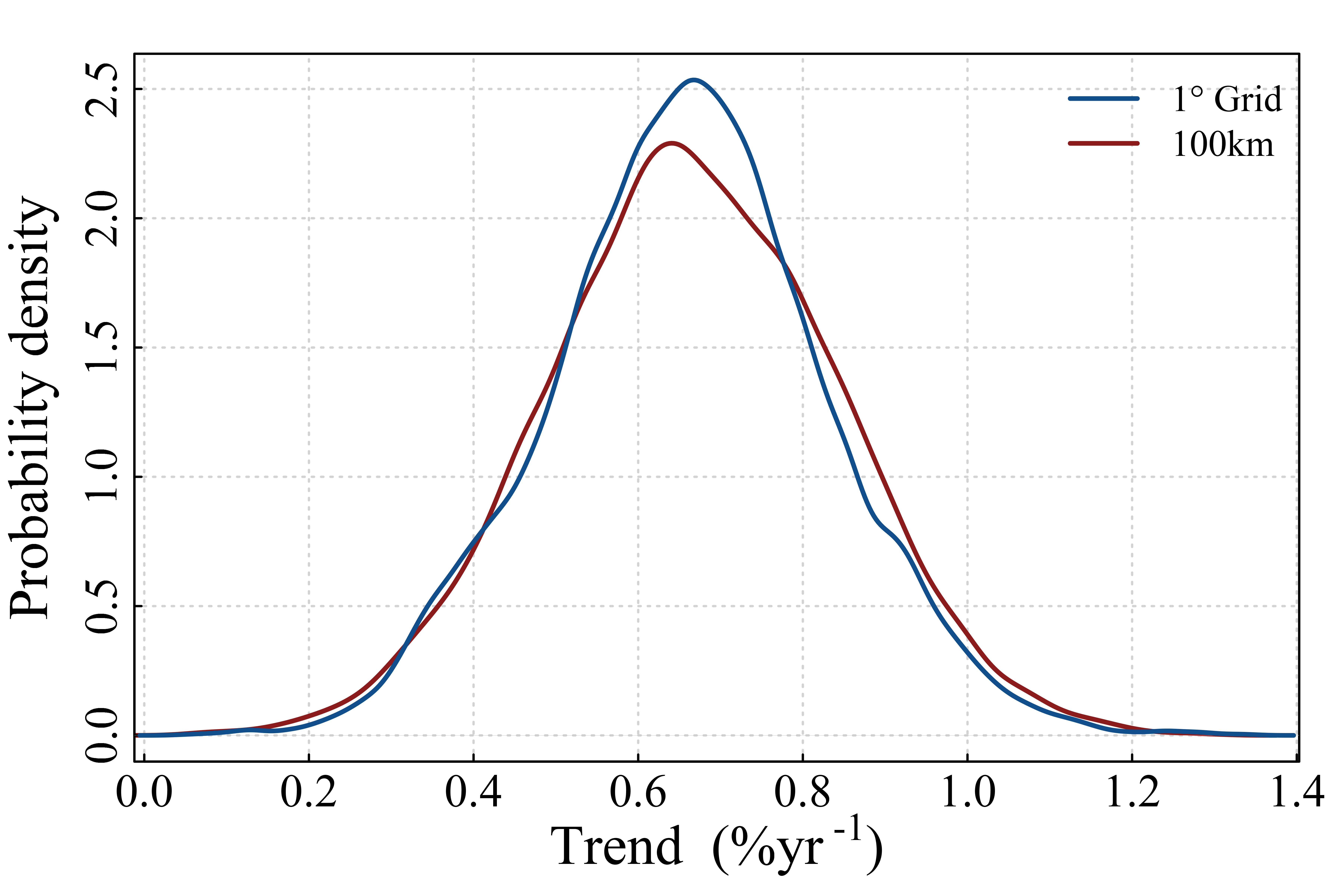


Figure S5. The effect of data gridding on posterior distributions of the trend estimate. The blue line indicates the distribution of trend estimates using vague priors and data gridded at a 1° equal resolution (as used in the rest of the manuscript). The red line indicates the distribution of trend estimates using vague priors and data gridded at a 100km equal resolution. Only minimal differences are seen between the two posterior distributions (summary values are listed in Table S5).
